# Ethical and Scientific Perspectives of Placebo-controlled Trials in Schizophrenia

**DOI:** 10.4021/jocmr2009.07.1247

**Published:** 2009-07-03

**Authors:** Yuval Melamed, Adiel Doron, Orit Stein-Reisner, Avi Bleich

**Affiliations:** a Lev-Hasharon Mental Health Center, Netanya, Israel, affiliated to the Sackler Faculty of Medicine, Tel-Aviv University, Tel-Aviv, Israel

## Abstract

Clinical trials for development of new medications are essential in all fields of medicine. The requirement for a placebo arm in pharmaceutical trials presents ethical and clinical dilemmas that are especially complicated with regard to mentally ill persons whose free choice and ability to provide informed consent may be questionable. On the other hand, we do not believe that this predicament justifies unconditional rejection of placebo use in psychiatry, when the investigational drug may ultimately provide substantial benefit for some patients. At the same time it is the psychiatrist's responsibility to insure that investigators are adequately trained to conduct clinical trials and that stringent regulatory committees supervise the scientific, clinical and ethical aspects of the trials.

**Keywords:**

Placebo-control; Schizophrenia; Medical ethics; Clinical trials

## Introduction

Clinical trials involving human subjects give rise to ethical and medico-legal dilemmas. Essential research of new drugs may potentially expose patients to ineffective medications or to placebo. The complexity of the issue increases when dealing with mentally ill patients for whom there is no known cure, and whose ability to provide informed consent to participate in clinical trials may be questionable.

In the absence of a consensus regarding criteria for placebo use Kotler and Witztum [1] claim that we are witnessing a growing controversy. This debate has practical implications for Ethics Committees (Internal Review Boards) when deliberating approval of participation in extensive essential psychopharmacological research. The issue has been addressed by psychiatrists in various countries [2]. We present the ethical considerations to be dealt with by the treating physicians and investigators.

## Declaration of Helsinki

The basic principle of the Declaration is that each and every patient is entitled to the best treatment available [3]. In 1996 it was determined that in all medical research, every patient, including those in control groups is entitled to the best available treatment, and that placebo may be used only in the event that there is no proven treatment available.

In the year 2000 a correction was added, which determined that the benefits, risks and effectiveness of new treatments must be weighed in comparison to the very best available treatment, and the use of a placebo arm should be limited to situations where there is no proven effective treatment.

In 2002 the World Medical Association (WMA) inserted a clarification that claimed that placebo-controlled trials could be ethical, even where proven treatments are available in the event that: there are scientific/methodological grounds for inclusion of placebo in the trial as an essential condition to prove efficacy of the drug [4].

## Research versus treatment

Medical treatment and medical research differ. The Physician's Oath, adopted by the General Assembly of the World Medical Association, (1948, amended 1968) pledges that "The health of my patient will be my first consideration". Naturalistic or case control study designs do not interfere with the patients' treatment regimens, and they are in accord with these principles of treatment. However investigations of new drugs are randomized controlled trials which require research methods that often contradict accepted standards of treatment.

Determination of dosage is in accordance with the research protocol, and may be influenced by additional agents including investigators, medical institutions, commercial and insurance companies [5].

An additional problem is that clinical trials often require a washout period during which the patient receives no effective treatment.

## Types of studies

A number of study designs can be used to prove treatment efficacy: placebo-controlled trials; active-drug comparison trials, control group in comparison of dosages, historical control groups and control groups with no treatment [6]. The latter two designs are not considered valid for psychopharmacological studies.

In placebo-controlled studies, or dosage comparisons, the goal is to emphasize superiority of the new drug, in any dosage, in comparison to placebo, or a lower dosage of the same drug.

In a trial with a control group receiving active treatment - the aim is to prove superiority of the new drug over the active comparator. It is difficult to expect a new treatment to be better than a recognized standard therapy during the various stages of the study, therefore many investigators would prefer the situation where studies that plan to show equal value or non-inferiority would be considered valid.

The research hypothesis that it is possible to differentiate between various treatments is called assay-sensitivity. This assumption is based on prior studies with active standard treatments, but it should be noted that the instruments used for measurement in psychiatry are often based on observation, self-report or interview and not on objective measures. Some medications that are in use and considered effective, have not always been proven as such in comparison to placebo. Thus, if we compare a new drug to these agents, we cannot scientifically prove that the new drug is effective, even if the trial reveals that it is of equal value to existing medications.

A placebo arm in a clinical trial enables investigation of assay sensitivity. When there is no placebo arm in a trial, it may be necessary to increase the size of the study group to prove the postulation of non-inferiority, thus complicating performance of the study. In addition, in such a case more patients will be exposed to an experimental drug whose efficacy has yet to be proven. Similarly, the smaller the gap, when comparing new and existing treatments, the larger the study sample needed in order for the difference to have statistical significance.

An additional experimental possibility which is used, for example in cardiology, is an add-on trial drug with a placebo-control [7]. This method (placebo-controlled add-on) reduces the dilemma of administering placebo, since all participants receive active treatment.

## Informed consent

Due to the potential discordance between research and treatment, the patient must be aware that he/she is being treated in the framework of a research protocol and he/she must provide written informed consent. Since the competency of schizophrenia patients to provide valid informed consent is questionable, stringent regulation is essential for the informed consent process involving this population.

Thought disturbances and cognitive impairments characteristic of schizophrenia may give rise to concern regarding the ability of the patient to provide informed consent [8] especially patients with chronic illness [9].

In addition, the psychiatric patient who is hospitalized for long periods of time may more readily agree to participate in clinical trials offered by his attending physician, because of his/her dependency on the hospital.

The Working Party on Research on the Mentally Incapacitated, London, discussed the issue [10] and determined that many people with mental impairment or disorders are able to consent to their inclusion in research provided care is taken to explain it to them. Patients competence should be evaluated using questionnaires designed especially for that purpose [11].However, there are those who believe that provision of informed consent neither protects the patient nor ensures an ethical basis for the study since the patients condition interferes with his/her ability to objectively weigh the various treatment options [12].

## Placebo controlled trials in schizophrenia

The need for new pharmacological treatments is based on the fact that some patients respond to currently available antipsychotic agents with only partial improvement, especially in all aspects related to negative schizophrenia symptoms. The existing treatment for this disease is effective mainly for positive symptoms and in delaying relapse of the disease in chronic patients. Moreover, the efficacy of antipsychotics in preserving cognitive functions has yet to be proven. In addition, the existing drugs, including second generation medications, have significant side-effects that may impair the health and quality of life of schizophrenia patients. In the formal sense there is no Gold Standard of care for negative symptoms, so it is necessary to engage in research that compares efficacy of the preparation to placebo [13].

In the United States, to gain Food and Drug Administration (FDA) approval, significant proof of the efficacy of the drug based on adequate controlled trials must be presented [14]. The majority of investigative new drug applications is based on the results of placebo-controlled trials [15]. The European authority parallel to the FDA approves drugs based on trials where the new preparation is compared to placebo and/or treatment with standard medication.

Positive response to placebo in schizophrenia has been shown to range from 3 to 26% in various studies [16]. Different patients respond differently to placebo, including those who apparently respond better to placebo, and it is probably not worthwhile to include those patients, if it is possible to identify them ahead of time [17].

## Difficulties regarding placebo control in schizophrenia and possible solutions

The main argument against placebo use is associated with the inherent difficulty of the physician to refrain from giving the patient effective treatment. This issue is difficult for every physician/investigator and especially for physicians who treat the mentally ill.

Absence of active treatment may cause long and short-term risk or harm. Early diagnosis and treatment has been shown to achieve the best possible results in reducing negative symptoms [18] or improving the chance for a longer remission [19]. Although some studies showed that even if the patients suffered exacerbation of illness after stopping active treatment, they returned to their basic state within a number of days or weeks with no long-term harm [20], however not all patients enter remission following episodes of exacerbation.

Thus, we believe that first episode patients should not be recruited to clinical drug trials unless the study focuses on treatment specific to this population.

The emotional state of the patients and the possibility of cognitive impairment raise questions regarding their capacity to provide informed consent for participation in research.

This can be avoided by routinely using questionnaires that specifically examine the patient's competency to participate in clinical trials [11]. In order to eliminate staff influence, the research staff should be distinguished from the caregiving staff, and an external physician may participate in the recruitment stages of the investigation.

Patients receiving involuntary treatment either in the hospital or in the community require special consideration regarding recruitment to placebo-controlled trials. It is questionable whether a patient who does not consent to hospitalization can voluntarily consent to participation in a pharmaceutical trial. Alternatively, there is the view that though a patient may oppose treatment and hospitalization, if medication is compulsory, he/she may prefer one treatment over another, or may agree to a specific treatment [2]. The authors believe that these patients should not be included in clinical trials, due to the sensitivity of the issue.

Other opinions suggest that patients recruited to placebo studies are not necessarily representative of the general patient population. They may be older, with a longer duration of illness and more willing to participate in clinical trials because a cure has not yet been found for their illness. In addition, Placebo-controlled trials have a higher dropout rate [21].

A significant problem in commercial studies is the financial aspect: Today, most clinical trials for new drug applications are designed and sponsored by pharmaceutical companies. Commercial companies are interested in placebo-controlled trials and the direct financial relationship between the company and the investigator may be problematic [22], and should be avoided.

The investigator must assure beneficence, a favorable balance between the potential benefit and harm of participation.

## Discussion

There is no known pharmacological cure for schizophrenia. Treatment provided by physicians is in accord with the current knowledge of the illness, and is dispensed to alleviate the symptoms and to improve the patients ability to function, and quality of life. Physicians will always hesitate to expose a patient to an unfamiliar agent when a known medication is obtainable. On the other hand, without medical research and clinical trials, we would not have the effective medications that we have today, which pending ongoing medical research, may indeed be replaced by more effective preparations in the future.

The increasing rift surrounding the use of placebo in trials for the development of new psychiatric medications, involves scientific, clinical and ethical issues. It seems that there is no single all encompassing approach either for or against placebo-controlled trials. Placebo use is problematic in medicine. Administering treatment which in fact may be a placebo alters the physician-patient relationship, especially when dealing with mental health patients suffering from schizophrenia. However, since there is no known cure for the disease, research is essential in this population, so it may be justifiable to encourage patient participation in clinical trials to advance medical knowledge in the field. In most cases the use of an active control in schizophrenia research is ethically and scientifically preferable [23]. This is not always possible.

It is the investigators responsibility to ensure that clinical trials are designed and conducted according to appropriate clinical and ethical principles and that each proposed trial is based on solid hypothesis according to which the new treatment will be more effective and/or have less side-effects than existing treatment.

In a clinically justified trial, when including a placebo control group, the size of the placebo group and duration of administration of placebo should be as limited as possible and the duration of the medication washout period should be as short as possible.

A balanced approach should allow for placebo use while examining the various aspects and delineating safeguarding restrictions accordingly [24]. The investigator should bear in mind that referring a patient to participate in a study is basically positive.

It should be emphasized that participation in clinical studies has benefits for the patient, such as increased monitoring and clinical care in the research setting where he/she is examined more frequently, intensified psycho-social services, and the opportunity to continue to receive the experimental drug free of charge following successful completion of the study.

Active quality control of clinical trials should be carried out by the trial sponsor or by their representatives [25]. Quality control begins from the stage of the presentation of the study protocol to the potential investigator for a feasibility review, and is complete when conclusions are drawn and findings are reported.

It is important to train young investigators in the methodology and guidelines for good clinical practice for management of clinical trials, with emphasis on the special issues involving psychiatric patients. Conducting trials in accordance with the International Conference of Harmonization (ICH/GCP) Harmonized Tripartite Guidelines for Good Clinical Practice [26] provides assurance of quality research and patient protection. In addition, Internal Review Boards and ethics committees are responsible for safeguarding the rights, safety and well-being of all trial subjects, with special attention to trials that may include vulnerable subjects.

## Conclusions

Clinical pharmaceutical trials are essential for the development of new medications in all fields of medicine, including psychiatry. The requirement for a placebo arm in pharmaceutical trials presents ethical and clinical dilemmas that are especially complicated with regard to mentally ill persons whose free choice and ability to provide informed consent may be questionable. The authors do not believe that this predicament justifies unconditional rejection of placebo use in psychiatry. Each case should be considered and evaluated for risks and benefits for participants and for the general patient population involved.

It is therefore important to require adequate training and certification for investigators and to provide stringent restrictions that will enable strict supervision over the scientific, clinical and ethical aspects of clinical trials.
